# Integrated Assessment of Artisanal and Small-Scale Gold Mining in Ghana — Part 3: Social Sciences and Economics

**DOI:** 10.3390/ijerph120708133

**Published:** 2015-07-15

**Authors:** Mark L. Wilson, Elisha Renne, Carla Roncoli, Peter Agyei-Baffour, Emmanuel Yamoah Tenkorang

**Affiliations:** 1 Department of Epidemiology, School of Public Health, University of Michigan, 1415 Washington Heights, Ann Arbor, MI 48109, USA; 2 Department of Afroamerican and African Studies, University of Michigan, 101 West Hall, Ann Arbor, MI 48109, USA; E-Mail: erenne@umich.edu; 3 Department of Anthropology, University of Michigan, 101 West Hall, Ann Arbor, MI 48109, USA; 4 Department Anthropology, Emory University, 1557 Dickey Drive, Atlanta, GA 30223, USA; E-Mail: carla.roncoli@emory.edu; 5 Department of Community Health, School of Medical Sciences, College of Health Sciences, Kwame Nkrumah University of Science and Technology, Kumasi, Ghana; E-Mail: agyeibaffour@yahoo.co.uk; 6 Institute for Development Studies, University of Cape Coast, Cape Coast, Ghana; E-Mail: eytenkorang@yahoo.co.uk

**Keywords:** artisanal small-scale gold mining (ASGM), subsistence agriculture, alternative livelihoods, “poverty trap”, ASGM policy, miner registration, West Africa, economic development

## Abstract

This article is one of three synthesis reports resulting from an integrated assessment (IA) of artisanal and small-scale gold mining (ASGM) in Ghana. Given the complexities that involve multiple drivers and diverse disciplines influencing ASGM, an IA framework was used to analyze economic, social, health, and environmental data and to co-develop evidence-based responses in collaboration with pertinent stakeholders. We look at both micro- and macro-economic processes surrounding ASGM, including causes, challenges, and consequences. At the micro-level, social and economic evidence suggests that the principal reasons whereby most people engage in ASGM involve “push” factors aimed at meeting livelihood goals. ASGM provides an important source of income for both proximate and distant communities, representing a means of survival for impoverished farmers as well as an engine for small business growth. However, miners and their families often end up in a “poverty trap” of low productivity and indebtedness, which reduce even further their economic options. At a macro level, Ghana’s ASGM activities contribute significantly to the national economy even though they are sometimes operating illegally and at a disadvantage compared to large-scale industrial mining companies. Nevertheless, complex issues of land tenure, social stability, mining regulation and taxation, and environmental degradation undermine the viability and sustainability of ASGM as a livelihood strategy. Although more research is needed to understand these complex relationships, we point to key findings and insights from social science and economics research that can guide policies and actions aimed to address the unique challenges of ASGM in Ghana and elsewhere.

## 1. Introduction

Artisanal and small-scale gold mining (ASGM) has contributed significantly to Ghana’s gross domestic product (GDP), making this activity an important source of employment and income for miners and their dependents. While ASGM represents a relatively small proportion (~10%) of Ghana’s annual gold mining production [1], it is a growing sector that affects the livelihood of increasing numbers of people each year. In 2011, approximately 245,000 ounces of gold extracted by ASGM activities were purchased by and then sold through two Ghana-based mining companies (Precious Minerals and Marketing Corporation and Asap Vasa). At the average annual 2011 price of U.S. $1568 per ounce [2], this represented about $386 million generated by ASGM, not including an unknown—but estimated to be substantial—revenue from sales through informal markets and non-traditional means. The following year, ASGM production rose by 43% to 357,493 ounces, which, at the 2012 average annual price of $1669 per ounce, represented about $597 million of ASGM gold, a one-year increase of more than 64% in market value ASGM production.

Globally, more than 100 million people depend directly or indirectly on artisanal mining for their livelihoods [3,4]. In Ghana alone, an estimated 1.1 million people directly work in ASGM activities, representing nearly two-thirds of the country’s total mining labor force [5,6]. Much of this activity is considered “informal”, “unregistered”, and illegal. The proportion of Ghana’s gold that is mined through ASGM has increased from 6% in 2000 to 23% in 2010 [7]. 

**Figure 1:**
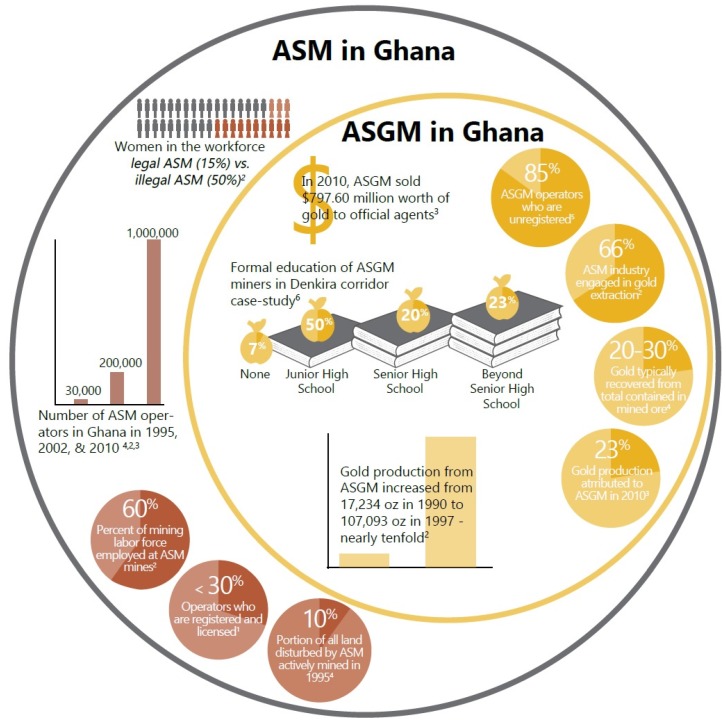
Graphic of estimated statistics available on ASM (artisanal and small-scale mining) and ASGM (artisanal and small-scale gold mining) in Ghana. Notes: **^1^** Aubynn [12]; **^2^** Hilson [13]; **^3^** Tetteh [7]; **^4^** World Bank [14]; **^5^** Hilson and Potter [15]; **^6^** Kessey and Arko [16].

Because of its economic benefit, ASGM is on the rise, in Ghana and globally (Figure 1). For individual miners and their families, engagement in ASGM is partly motivated by the need to generate cash to purchase necessities of daily life. Under conditions of unemployment and poverty, where most alternative work involves low or no pay, participation in ASGM, legal or not, has become a primary means of survival for many [8,9,10]. The starting point for understanding the microeconomic drivers of ASGM is recognizing that for many it represents work intended to meet livelihood needs, most often driven by lack of alternative sources of income. Yet any proposals to formalize ASGM through more extensive registration programs or encourage alternative livelihood options have to be realistic and sustainable if they are to be accepted [11]. 

### 1.1. Objective

This review presents findings from research conducted by the Social Sciences and Economics Workgroup of the “Integrated Assessment of Artisanal and Small-Scale Gold Mining in Ghana”. It constitutes one of three workgroup synthesis summaries [17,18] that frame this special issue of the International Journal of Environmental Research and Public Health [19] (http://www.mdpi.com/journal/ijerph/special_issues/asgm).

The over-arching policy-relevant questions that guide the Integrated Assessment include: What are the causes, consequences and correctives of small-scale gold mining in Ghana? More specifically: What alternatives are available in resource-limited settings in Ghana that allow for gold-mining to occur in a manner that is safe for human health [17] and environmental health [18] without affecting near- and long-term economic prosperity? Given the complex and global nature of ASGM, this Integrated Assessment (IA) provides a framework [20] that structures the analysis of economic, social, health, and environmental data, and co-develop best-practices, evidence-based solutions with pertinent stakeholders. An IA evaluates the causes and consequences of issues, usually with environmental, economic, and social dimensions, in an integrated and comprehensive manner, and then considers options to address them. This approach is often used when tackling controversial or poorly understood problems that lack a priori consensus on definition of the problem or question that needs to be addressed [20,21]. Typically, IAs will first define the policy-relevant question, the document status and trends, next describe the causes and consequences of those trends, and finally identify desired outcomes and policy options [22]. Often, an IA may also evaluate likely outcomes of each option, provide technical guidance for implementation, and assess uncertainty. Each topic that we address below considers: Causes, Status and Trends, Consequences, and Certainty Analysis.

In particular, in this analysis we examine the historical context and the social and economic drivers that underlie and perpetuate ASGM in Ghana, and shaped the contemporary situation. The evidence that we present comes from our own original research and our analysis of previously published reports and documents. Indeed, all coauthors have been working on these issues for many years, thereby contributing an understanding of context and culture that is not necessarily evident from our published reports. The ultimate goal of the analysis is to identify response and policy options associated with ASGM in Ghana that may lead to improved human health and well-being [23,24,25,26]. To ensure their viability and sustainability, these options must be relatively inexpensive, sustainable, low-tech, health-promoting, as well as socially acceptable and equitable, while maintaining or improving the standard of living of people who currently are involved in ASGM activities in Ghana [5,10,27,28,29].

### 1.2. Limitations and Assumptions

This summary builds on the past work of many scholars who have produced data-driven analyses, careful interview-based evaluations, thoughtful interpretations, and insightful recommendations aimed at understanding and improving the lives of people working in ASGM throughout the world. Our work aims to distill key findings from previous research that throws light on specific ASGM issues in Ghana. We do not seek to produce an exhaustive summary of world-wide research on ASGM or to relate the results of those studies that we do consider in their entirety. Rather, our report integrates and contrasts relevant materials from previous work with key findings from our own research, though the latter have been condensed and not included in full, since some of the results have not yet been published. Published literature was identified using standard databases of scholarly publications, combined with internet searches of “grey literature” from government, NGO, and other websites. We have not attempted an exhaustive review of all relevant information, but have identified and integrated pertinent studies and analyses. Finally, this report does not include information on the direct environmental [18,30] or health [17,31] impacts of the mining process, as the latter are the focus of other IA reports in this series.

## 2. An Assessment of Social and Economic Issues

Here we present the main social and economic drivers of and outcomes from ASGM in Ghana, and their positive or negative impacts on the well-being of individuals, families and communities. This assessment of current scientific knowledge aims to contribute to building consensus and guiding decision-making centered on policy response options. The goal is to identify socio-economic problems, to understand their context and histories, and to point to possible options for sustainable change. Fundamentally, ASGM can be considered for its microeconomic drivers and consequences (e.g., its role in contributing to basic human needs) as well as the broader, society-wide, macroeconomic processes with which it is associated (e.g., by contributing to economic growth through capitalization of small businesses or agricultural intensification). We have outlined some consequences of ASGM that should serve to prioritize the most important challenges identified in the previous section. We focus on impacts that are clearly evidence-based (*i.e.*, high likelihood of this being a problem), that span and link multiple sectors and issues, and that explain the response options proposed in the companion report by Basu *et al.* [17] on human health.

### 2.1. Poverty and Livelihood: Microeconomic Effects and Needs

Artisanal and small-scale mining activities provide an important source of livelihood for both proximate and distant communities [24,25]. In such communities, mining serves as both a means of survival for impoverished farmers and as an engine for small business growth and agricultural expansion, complementing national micro-, small-, and medium-size enterprise projects. Indeed, Okoh and Hilson [32] have analyzed the strong ties between subsistence agriculture and ASGM in rural Ghana, arguing that such mining represents an important means of income diversification for many farmers, making it important to simultaneously address both agriculture and mining issues while improving people’s lives. For example, in areas with distinct dry and rainy seasons, ASGM provides farmers with income-generating opportunities during the dry season, while subsistence agriculture occupies them during the rainy season. Although ASGM employs a significant proportion of the work force in many localities where they operate, the informal nature of this employment and lack of official records makes it difficult to quantify. 

**Figure 2:**
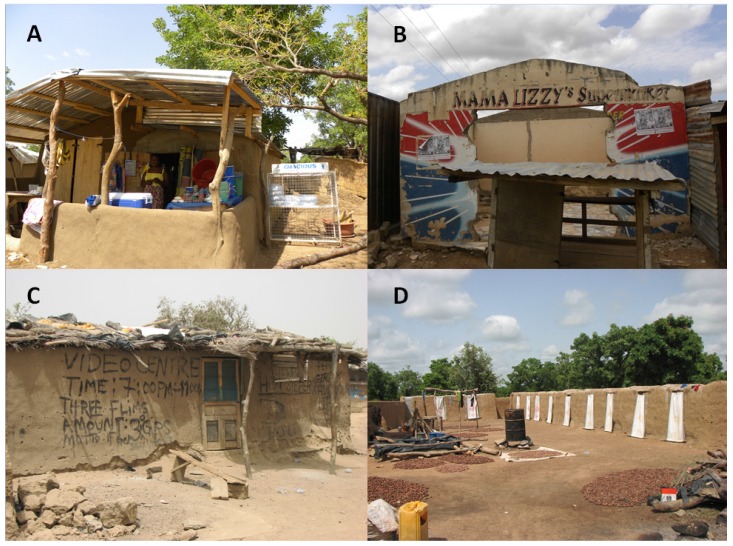
Examples of income generating activities linked to ASGM at our study sites in Ghana: (**A**) Shop selling food and water (Kejetia mining site, Talensi District, photograph Mark L. Wilson), (**B**) Outdoor “Supermarket” (Kejetia mining site, Talensi District, photograph Mark L. Wilson), (**C**) Video Centre for viewing entertainment (Kejetia mining site, Talensi District, photograph Elisha Renne), (**D**) Bathing stalls for short-term rental (Kejetia mining site, Talensi District, photograph Elisha Renne).

The contribution of small-scale mining may also produce domino effects if enhanced revenues and infrastructures are reinvested in the same locality [33]. ASGM drives demand for goods and services through the generation of purchasing power. For instance, it is common knowledge that in small-scale mining communities, both men and women engage in income-generating activities such as the selling of food (Figure 2), of mining-related products, and of gold and other minerals. They are also creating firms that are subsidiaries or complementary to small-scale mining. For example, in one small-scale gold mining concession in the Upper East Region, pumps (and generators to run them) were purchased to remove water from deep mining. 

However, there are negative social and environmental consequences of ASGM as well. Although not strictly a microeconomic effect, ASGM communities may experience elevated levels of aggression and violence between resident miners and newly-arrived settlers (sometimes from other regions) or foreign large-scale mining operations [25]. It is also possible to see displacement of farmers and others involved in community development emanating from competition for land, water, and other natural resources. Also, according the Yakovleva [34], the marginalization of agriculture has forced some rural women to enter into ASGM, where they are usually engaged in gold mining activities with low returns. The role of small-scale mining as the backbone of some local economies is seen to facilitate the development of complementary, sustainable, revenue generating activities, which may serve as a source of finance for local investors to run their small businesses [33]. Strong evidence suggests that ASGM is being used to produce income to provide for basic needs such as food, children’s education, health care, clothing, and shelter [4], most being important components of the Millennium Development Goals (MDGs). However, the benefits may not be spread equitably among men and women in a community and are context-dependent, as gold-bearing rock in some mining sites may become increasing difficult to access. Studies in other African settings have shown that ASGM may actually lead to a decline in income. People may invest time and money in purchasing equipment without obtaining sufficient gold profits, as was reported in Sangha Trinational Park at the intersection of Cameroon, Republic of Congo, and the Central African Republic [4]. Thus, artisanal mining is a risky business, with no guarantee that poverty will be reduced [35]. 

#### 2.1.1. Causes

Microeconomic reasons to take up mining, particularly with the high price for gold, include the inability to find other work, the inadequate pay of other available jobs, and the low educational status of many, which limits access to better paying jobs [5,11,36]. The issue of low wages of alternative jobs, in relation to the potential economic gains through mining [37], is particularly important and deserves careful attention, particularly in areas where the work of small-scale gold miners without registered concessions has been prohibited by district assemblies acting under the directives of the federal Inter-Ministerial Task Force Against Illegal Mining, established in 2013 [38]. There is debate regarding the relative importance of “push” *vs.* “pull” factors in shifting people to undertake ASGM [28]. Circumstances that may "push" people from subsistence farming, for example, include local population growth, diminished soil fertility or agricultural productivity, decreased cash-crop profitability, inadequate access to farming inputs, market failures or natural disasters and climate extremes. Among possible “pull” factors that draw people to ASGM are higher return on labor, lower risk in economic activities not centered on agriculture, generation of cash to meet non-food needs, and other economic opportunities associated with development and urbanization [28]. Although there are “gold rush miners” who are lured by prospects of striking it rich, they seem to be few and atypical. Indeed, most evidence indicates that the growing number of people who are participating in ASGM do so out of sheer poverty and lack of alternatives [39,40]. In other words, most of these people are “pushed” into ASGM out of necessity, rather than being “pulled” by a “get rich quick” prospect [28]. In certain settings, those coming from families with long involvement in mining may continue mining because of cultural and social attachments to this lifestyle and its acceptance by the community [36]. 

**Figure 3:**
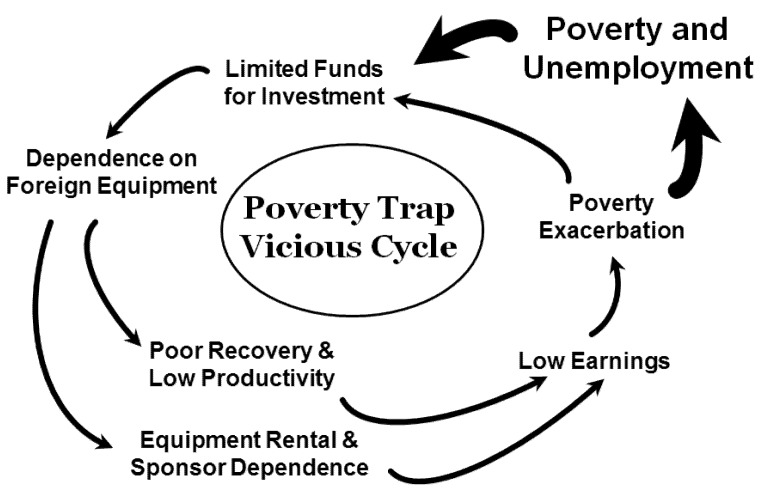
Illustration, modified from Hilson [35] after UNECA [41], of the “Poverty Trap” faced by ASG miners and their dependents who have little resources to invest, depend on inadequate equipment that leads to low productivity and little earnings, which, in turn, exacerbates poverty and dependence on ASGM.

A 2012 report on ASGM by Hilson [35] in the Talensi-Nabdam District of the Upper East Region of Ghana has developed this theme of mining as a “poverty trap”, which is illustrated in the interactions of Figure 3. This “vicious circle” of dependence involves un- or under-employed people or subsistence farmers who shift activities to ASGM, often moving to new locations where it is possible or already being practiced, with the hope of earning some income. Having little or nothing for investment in more efficient technology, they labor at dangerous work that typically produces minimal productivity and little wealth accumulation. These miners and their families may also depend on mining and processing equipment from others for their ASGM work, further indebting them to others, keeping them in a “poverty trap”. 

#### 2.1.2. Status and Trends

**Figure 4:**
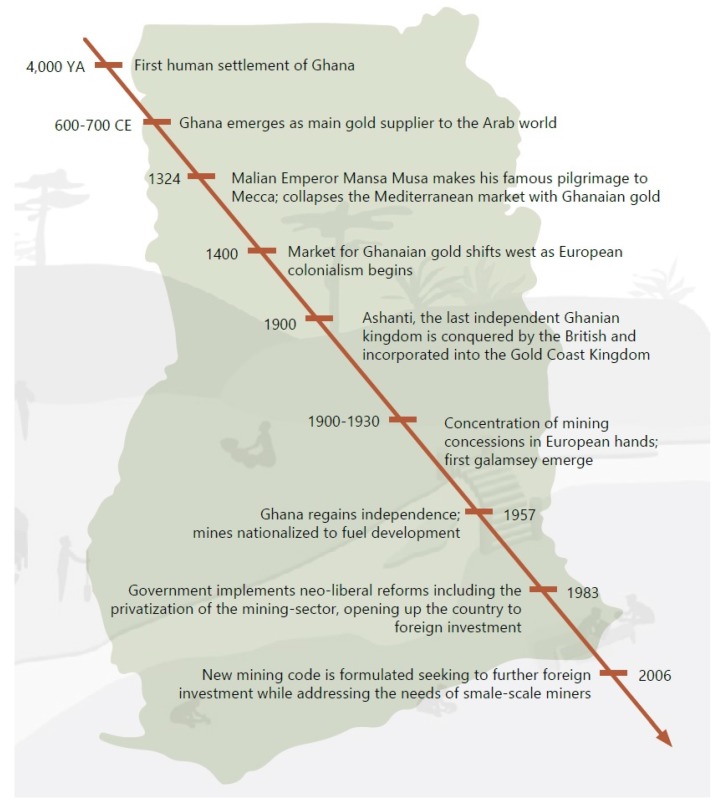
Timeline of Ghanaian mining history.

The history of ASGM in Ghana has been reported through various studies and reviews [13,42,43], (Figure 4). For example, according to the University of Ghana historian Ababio Ofosu-Mensah [24], gold mining was practiced in the Adanse and Akan Kingdoms in south central area of present day Ghana, for over 1000 years. Gold served as a key resource for a political system of kingship. By the 6th and 7th century A.D. Ghana had already emerged as one of the largest gold producers in the world, supplying the majority of gold to the Arab world via Saharan trade routes. Mining was mostly conducted in the Akan states of Asante, Denkyira, Akyem and Wassa, with gold emerging as the primary method of payment for taxes and tribute. Traditional authorities played an important role in regulating mining activities, protecting communal water sources and settling disputes between miners. Mining was conducted both seasonally and yearly, and relied on a number of techniques ranging from panning, to shallow pit and deep shaft mining, similar to operations today. In many ways there exists continuity between the *galamsey* (informal miners) of today, and traditional Ghanaian mining. Indeed, as Ofosu-Mensah has noted [24]. “Despite the existence of large multinational mining firms such as ANGLOGOLD Ashanti [and] Newmont Ghana Gold Company Ltd.…pre-colonial gold mining has retained its vigour”.

A number of historical and contemporary studies by colleagues and ourselves have helped shed new light on the complex social and economic dynamics in the eastern, central, northwestern, and northeastern areas of the country. A recent study on gold mining in Nangodi, Upper East Region, documents the intersecting gold-mining histories which have linked residents of this small rural community with successive political regimes, changing mining laws, and the vagaries of the global economy reflected in fluctuating gold prices.

ASGM in Nangodi, which was also the site of the first deep shaft mine in northern Ghana [44] has been influenced by men involved in large-scale mining activities in southern Ghana [45], with successive waves of gold mining practices affecting the lives of Nangodi residents—economically, politically, and in terms of their health, nutrition, and environment. As elsewhere in Ghana, while Nangodi residents have benefited economically from gold mining, they have also experienced health and environmental consequences of ASGM, underscoring the vulnerability as well as resiliency of such gold mining communities [46]. More recent research carried out in 2014 in Nangodi suggests that District Assembly measures to prosecute illegal miners have reduced gold mining there [38].

In another study from Bole District in the Northern Region, Samuel Ntewusu, a research fellow at the University of Ghana and member of our research group, analyzed how historical mining from the 1800s influences today’s ASGM activities in the district (Samuel Ntewusu, personal communication). Bole gold was exchanged with other regions of Ghana and Europe (1910s and 1920s) in the interests of the British colonial rulers. In the 1960s, after independence, private firms experienced low returns and poor performance, thus forcing them to leave this area in the 1980s and 1990s, and opening up the region to contemporary illegal mining. Yet current ASGM in Bole District differs from earlier practice because in addition to the negative environmental consequences of surface gold mining, there has been a rise in the negative social consequences of ASGM, which include armed robbery, prostitution, illegal weapons, drugs, and counterfeit currencies. Nevertheless, Bole District has grown demographically and economically, with increased trade and transportation and an ethic of greater self-reliance. Hilson *et al.* have also noted the economic benefits of ASGM in Bui, in Bole District, for farming families [47]. Furthermore, while Ntewusu notes that many traditional institutions have nearly disappeared (e.g., chieftaincy, traditional African religions, warrior cult (*mbonwuras*)), others have reappeared to address of some of these social problems. Ntewusu’s work demonstrates the complex, multi-level, historical, social and economic interactions surrounding evolving ASGM (Samuel Ntewusu, personal communication). 

Work by other members of our group (Ofosu-Mensah, submitted) [48] has evaluated gold mining in the historical Akyem Abuakwa Kingdom, in the Eastern Region of Ghana, which dates back for centuries. The Abuakwa goldfields were one source of gold to the European market before and during the colonial administration of the Gold Coast. The introduction of modern mining techniques in Ghana during the 1890s enabled European mining companies to attract miners and gain control over the mining industry [26]. Indigenous Ghanaian miners, however, persisted then, and their activities survive today in Abuakwa, Eastern Region. This study examined the consequences of both large- and small-scale mining in the Akyem Abuakwa state. Ofosu-Mensah notes that the economic and political power of traditional kingdoms in southern Ghana was related to their conquest of gold-bearing land in those areas [26]. Allodial rights in Abuakwa lands were vested in the Okyenhene, the Paramount Chief, who headed his sub-chiefs caretakers of the land in the control of gold mining and revenue. This gold wealth enabled the Okyenhene’s personal affluence and supported the making of gold jewelry which added to festivity and grandeur of state occasions such as the Ohum and Odwira festivals. However, this situation changed with Abuakwa chiefs’ financial decline in the late 1890s and early 1900s, as a result of the introduction of legitimate trade, the monetization of the economy, the increased demand for labor in other sectors such as industrial mines and cash crop production, as well as employment in colonial government. While these changes affected gold mining in relationship to chieftaincy institutions in Akyem Abuakwa communities, an understanding of its gold mining past provides the basis for future analyses of the dynamics of small-scale gold mining in this area in the late 20th and early 21st centuries, particularly the practices of galamsey mining in relation to traditional chiefs in this area.

#### 2.1.3. Consequences

ASGM creates various potential economic opportunities, advantages and benefits, as well as instability, risk and impoverishment, all of which are part of any production process. Some of these potential gains and hazards are particular to ASGM. At the individual level, year-round use of child labor and reduced attendance in schools [29] are likely to lessen opportunities for subsequent economic improvement later in life. Women represent about 15% of those employed in legalized ASGM activities, but perhaps as many as half of people working in Ghanaian *galamsey* (informal) mines [13,23,49] are women, who produce income directly for themselves or their families. However, many women see ASGM work as means of raising capital to start businesses that will allow them to quit gold mining work which they see as endangering their health and to move back to urban centers such as Accra [35]. Yet as many scholars have argued, miners and their families often end up in an economic “poverty trap” (Figure 3) in which people engaged in ASGM activity, involving low-tech, labor-intensive efforts, face mounting debts that prevent them from shifting to alternative employment that might be equally or more beneficial, economically and socially e.g., [29,35,50,51]. Little income, lack of investment opportunities, low productivity, and inadequate technological capacity all exacerbate one another to “trap” Ghanaian miners whose original goal was to enter this activity as a means of escape from poverty and lack of other opportunities (Figure 3).

#### 2.1.4. Certainty Analysis

Available evidence for Ghana strongly suggests that the principal microeconomic reasons that most people take up ASGM involve “push” factors aimed at trying to meet subsistence needs. There is less and conflicting information to suggest that the “pull” of a “get rich quick” perspective is important to very many people [34,35]. This probably varies across different regions of Ghana, however, there are no national comparisons to evaluate the relative importance of these drivers. There is moderate certainty (high plausibility) from peer-reviewed, published evidence that economic insecurity and inability to meet basic needs explain why most people initiate or continue in ASGM [52]. These and other observations also suggest with high plausibility that most families entering into ASGM end up in a “poverty trap” in which simple extraction methods, little investment, few alternatives, and lack of education combine to keep these families in poverty and dependent on this employment for survival.

### 2.2. Macroeconomic Effects, Policies and Programs 

The macroeconomic issues surrounding ASGM are multi-level and complex, not only in Ghana but in most other countries where it is practiced. As context, the ILO estimated in 1999 [53] that 13 million people throughout the world are engaged directly in small-scale mining activities, the majority of whom are in developing countries. That number is now estimated at more than 20 million [3]. In addition, the livelihoods of 80–100 million people are affected by small-scale mining. For instance, in China, it is estimated that ASGM provides employment for between 3 million and 15 million people [33]. The ILO asserts that the contribution of ASGM to mineral production is significant, accounting for approximately 15% to 20% of the world’s nonfuel mineral production. Compared with the large-scale mining sector, small-scale mining provides large volumes of various minerals, equaling or exceeding that produced by large mines, although little evidence exists on the exact volumes [33]. Despite the low levels of production achieved at an individual level, the large number of miners involved produces an aggregate that could be quite enormous. In Indonesia, for example, the total production of tin by the small-scale sector is said to equal to that of large-scale production [33]. 

The role of ASGM in the balance of payment of most countries may be seen in its contributions to the mining sector as a whole. However, due to poor or absent records in the informal sector, including ASGM, the exact magnitude remains unknown. In 2012, for instance, the mining industry contributed 27% of the Government of Ghana’s tax revenues, with gold export receipts amounting to more than $5.6 billion from 4.3 million ounces [54]. However, Ghana is expected to realize reduced gold production due to the difficulties faced by the ASGM sector [54]. 

In terms of fiscal impacts, some of the direct benefits from ASGM include access to finance and direct subventions (for the registered concessions), while indirect benefits involve, for example, taxes and technical assistance [55]. Apart from passive surveillance of disease prevalence in the areas of ASGM sites [56] and surveys [57], information is very scanty on the effects of ASGM on health status among Ghanaians measured by indicators such as disability-adjusted life years (DALYs) or quality-adjusted life years (QALYs). 

The reasons for the popularity of ASGM in most developing countries are manifold. Along with the recent rise in gold prices to over $1000/oz. since 2009, declining value of many export crops, loss of agriculture input subsidies (e.g., fertilizers), and changing rainfall patterns have forced individual small-holders to supplement incomes through non-farm activities such as ASGM [37]. One strategy to address this is to enhance support for agrarian-orientated activities to make this more economically sustainable. Such “re-agrarianization”, however, is not a simple panacea, as Banchirigah and Hilson [58] have argued, since the income generated by gold mining is often more remunerative More generally, “livelihood diversity” is often lacking in settings where ASGM has become a major source of income [28], resulting in few other employment alternatives to mining. 

#### 2.2.1. Causes

Recent macroeconomic development activities in sub-Saharan Africa have been based more on *laissez-faire* economic theory than on central planning [10,59,60]. Interactions with other sectors of the economy, such as large-scale gold mining, agriculture, and manufacturing, as well as government policies concerning taxation and land tenure, play important roles in determining access to and development of ASGM opportunities. Traditional land tenure systems in much of sub-Saharan Africa (SSA) are being challenged by policy changes reflecting influences from the global economy and emerging complexities within local socio-economic contexts [61]. In northern Ghana, for example, these influences appear to have resulted in “growing inequalities in access, control and ownership” of land, suggesting the need to redefine land rights so that they are “negotiated by a participatory process and regulated by both state and traditional institutions” [61]. Improved sustainability of ASGM in general [57], and specifically in Ghana [27] in terms of miners’ health and environmental impact, will require a synergistic approach involving multiple stakeholders deciding on the organization, regularization, and education of ASGM communities. 

During the economic recovery period of the 1980s, mining was identified as one of the strong contributing sectors that could help to revive the economy [57]. However, several problems with the artisanal small-scale gold mining sector were identified, such as the sale of ASGM gold on the black market, which was sometimes smuggled out of the country, and the indiscriminate, environmental damage done by illegal artisanal small-scale gold miners [46,48]. This situation contributed to the establishment of a committee to examine these problems and clarify its status within the mining sector. In 1989, three laws were passed which affected ASGM in Ghana: (1) the Small Scale Gold Mining Law (PNDCL 218), the Mercury Law (PNDCL 217), and the Precious Minerals Marketing Corporation Law (PNDC Law 219) [62]. Nonetheless, environmental problems associated with ASGM emerged, affecting land, forests, and waterways. Several international donors, such as the Netherlands and the World Bank, have contributed funds to address these problems; for example, the World Bank instituted a project, entitled Natural Resources and Environmental Governance Technical Assistance in 2013, that included the establishment, training, and equipping of District Mining Committees [63]. Yet the lack of registration of miners and the transitory nature of ASGM have confounded these efforts.

More recently the Ghanaian government has sought to address these problems through a presidential directive. In the second phase of the work of the Inter-Ministerial Task Force Against Illegal Mining (beginning in 2014), the task force intends to institute a program to register small-scale gold miners and to involve them in the reclamation of mined land [38]. In rural communities of Ghana, however, Bush [64] has argued convincingly that non-registered galamsey mining is an important source of livelihood that is being threatened through government regularization and criminalization. Since the fundamental problem of rural poverty is not being addressed, registration of small-scale gold miners only increases the vulnerability of those relying on ASGM, particularly women. Registration often entails literacy, access to registration centers, and application fees, requirements that discourage small-scale women gold miners from participating in this program.

Internationally, the Minamata Convention on mercury pollution has a stand-alone article to address ASGM (Article 7; Annex C) [65]. Notably, countries such as Ghana with significant ASGM sectors are required to develop environmental and public health strategies for ASGM communities, and take specific measures to protect vulnerable populations such as children and women of childbearing age. The Convention also encourages Parties to cooperate in education, outreach and capacity-building initiatives specific to ASGM (Article 7.4B) [65], as well as engage in general strengthening of public health measures to address mercury pollution (Article 16) [65], especially protection of vulnerable groups (women and children; Article 16.1A) [65] with specific mention of strengthening of institutional and health professional capacities (Article 16.1D) [65]. To help facilitate with the implementation of the Convention, a number of donor organizations have started to fund efforts in many low- and middle-income countries. One challenge is to apply the Minamata Convention guidelines in a manner that recognizes the challenges faced by marginalized mining communities, addresses livelihood insecurity, and develops polices that consider fairness and equity [66].

#### 2.2.2. Status and Trends

Macroeconomic Structural Adjustment Program (SAP) policies have been implicated in maintaining, even expanding, poverty within many sectors of Ghana's subsistence economy [10]. Indeed, SAP policies may be partly responsible for expansion of ASGM, and possibly marginalizing its participants [10]. By encouraging the expansion of large-scale gold mining through creating an attractive climate for foreign multinational investment in mining, thousands of people who were already practicing ASGM were displaced, thus exacerbating conflicts. Implementation of “procedurally complex and bureaucratically unwieldy regulations and policies” for ASGM have not favored miners in this sector [10]. 

Evaluating the diverse economic impacts of mining in general, and ASGM in particular, is challenging, and involves deciding which of the various metrics are to be used, at what societal level they occur and function, and how to measure and evaluate the direct and indirect pathways of influence [67]. Despite some differences among sub-cultures and environments in Ghana, ASGM shares many important similarities in the economic drivers and impacts that cross contexts of cultures, ecologies, geographies, and political or national differences [68]. Both the economic benefits and risks of ASGM, for individuals/families and for the Ghanaian economy, are considerable. Indeed, it is important to recognize that “(p)overty is multidimensional and the approaches to reduce it have to be similarly multidimensional: political, economic, social and environmental” [4]. Poverty reduction at a community level may result from ASGM, but the policies for this are not simple to implement [9]. As the mining sector develops and regulations/policies evolve, the issue of economic sustainability of ASGM are critical to evaluating the long-term impacts [69]. 

A study by Akabzaa and Darimani [70] explored the environmental and health impacts of ASGM on the land and people living in the area around Tarkwa, in the Western Region of Ghana. They documented degradation and loss of agricultural land and biodiversity, as well as air and water pollution. Additionally, malaria, upper respiratory tract diseases, especially pulmonary tuberculosis and silicosis, skin diseases, as well as injuries and accidents are associated with gold mining activities [17]. They conclude that while there have been economic benefits resulting from gold mining profits, the economic consequences for the environment and health have not been included in these calculations. The report concludes that mineral policy reforms in Ghana have contributed to an enormous increase in mining investment, but there has not been a corresponding review of environmental policies to take account of the damage caused to the environment and to sources of livelihood. Similarly, the increase in mining investment has resulted in a significant increase in gold production and the generation of external earnings. However, the wealth generated does not benefit either the national economy or communities located near the mines. The consequences have been a deepened crisis of health and environmental sustainability, social upheavals, and economic deprivation [70].

#### 2.2.3. Consequences

As outlined in the Introduction to this article, the underlying “cause” of ASGM is poverty and unemployment [28]. Low standards of living and the difficulty of working-age people to produce livelihoods for themselves and their dependents is a fundamental reality that motivates most to participate in ASGM. Some people whose basic needs are being met may shift to ASGM if they believe that it might lead to economic advancement, and possibly for asset accumulation, investment, and longer-term security. The macroeconomic limitations on other opportunities tend to limit options for employment and productivity in other sectors, particularly for the rural poor. Policy to enhance education aimed at providing new skills for workers, public health infrastructure, and expansion or development of traditional production sectors that are socially useful (e.g., agriculture, manufacturing, transportation) are likely to reduce the tendency of people to adopt ASGM as a sole recourse.

#### 2.2.4. Certainty Analysis

Considerable evidence points to the macro-economic contributions of ASGM to Ghana’s economic performance metrics, although the true magnitude of impact is difficult to quantify because of the extensive unreported and unrecorded galamsey mining. Indeed, even that which is reported is considered incomplete and of uneven quality. Other evidence is strong that the macroeconomic drivers underlying much of Ghana’s ASGM activity involve the paucity of gainful opportunities in other sectors that people face, forcing many to complement inadequate employment with illegal and dangerous mining. There is uncertainty, however, as to whether increased regularization would improve the livelihoods and living conditions of ASGM miners and their dependents. In addition, there is uncertainty as to whether the new mining laws will produce the stated goals, for example, of clarifying land rights, educating miners and their families, reducing conflicts and distributing taxation more equably among miners. The uncertainty of macroeconomic policy impacts exists against a backdrop of high certainty of human [17] and environmental [18] health risks. 

### 2.3. Legalization, Formalization and Enforcement 

A major challenge to improving productivity, increasing economic contributions, and enhancing wellbeing of people linked to ASGM is developing and enforcing rules around its practice that miners feel are in their interest and that they will support. This section discusses these challenges.

#### 2.3.1. Causes

ASGM in Ghana has historically been ignored, criminalized, and poorly regulated, leading to dangerous and difficult working conditions, as well as risk of criminal activities and assault [71]. ASGM activities are historically rooted in sub-Saharan Africa more generally, making efforts at formalization difficult [72]. The U.N. Economic Commission for Africa (2011) [6] has recently recognized that while there are adverse consequences to informal ASGM activities that are not regularized, the acquisition of mining rights by small-scale miners may be complex and governments may lack realistic plans for enhancing institutional capacity. 

The U.N. Economic Commission for Africa [6] thus underscored the need for governments to assist miners in shifting from informal mining to sustainable, registered activities, legally assure right-holders of sufficient land, and that there be land tenure security. Mining concessions for small-scale gold mining are often not clearly delineated, and illegal miners lack support to become regularized [27]. Mining tenure rights for ASGM needs to be addressed if those who practice small-scale mining are to become regularized/formalized in a sustainable fashion [73]. The ASGM subsistence trap becomes inescapable if miners cannot reinvest financially, apply new knowledge, and increase physical capital (equipment and land). The incentive to do this requires a “secure tenure” that is “simple, accessible, transferable, enforced and taxed at a level that does not give negative incentives…” [73].

#### 2.3.2. Status and Trends

Banchirigah, who studied ASGM in Noyem in the Eastern Region of Ghana, argues that such mining communities are often bound to their operations, having made considerable investments in equipment; others have few work options which are as remunerative, which helps explain why efforts at either regularization/formalization or developing alternative livelihood projects have mostly proven ineffective. 

This situation is not new, and has been documented by Tenkorang [52] who examined the history of the area now known as the Asutifi District of the Brong Ahafo Region of Ghana, where, dating back to the late 1800s, the colonial government attempted to monopolize gold mining in the Gold Coast. Colonial policies and laws weakened traditional authorities’ control over access to land and criminalized small-scale mining. In the latter part of the 20th century, the independent Government of Ghana realized the loss of revenue to neighboring countries from illegal trade in precious minerals and created a department within the Minerals Commission which was responsible for licensing, regulating, and supporting small-scale mining in Ghana. Despite this, many small-scale miners have not regularized their activities through obtaining mining concessions and are therefore operating illegally. This situation raises the question of how these miners gain access to the lands and who regulates their activities. The study of Asutifi District suggests that traditional rulers should be involved in this process [32].

#### 2.3.3. Consequences

The present system for formalizing ASGM does not address the new reality of this sector’s activities [43]. The recent rapid growth in ASGM, as well as incomplete and possibly inappropriate policies to regularize and reform the ASGM sector, means that the current formalization system may no longer be appropriate for the actual activities of the sector today. Registered and unregistered miners/owners operate in an intertwined and interdependent manner such “that the perceived dichotomy of formal and informal actors in the sector does not actually exist” [43]. Indeed, it seems that the apparent dichotomy between formal and informal activities does not really exist. Rather, the sector has developed into a “group of semi-formal sectors operating with varying degrees of legal registrations” [43]. In this regard, the U.N. Economic Commission for Africa [6] acknowledged the paucity of accessible institutional, technical and financial support for ASGM activities to designate and allocate areas for ASGM. Indeed, there is often lack of regional and international cooperation to address the challenges of conflicts surrounding mineral rights. Leniency and inadequate law enforcement means that most ASGM operates without much effective government control.

Many of the potential government approaches to formalizing, and legalizing ASGM activities identified from studies in Indonesia by Spiegel [74] seem quite relevant to Ghana. The government might simply ignore the issue (do nothing), but this would allow for problems to continue or exacerbate, and would give the impression that the government is ineffective. A second option would be to close down all unlicensed operations through criminalization, which reduce certain environment and health impacts in the short-term, but would have serious economic impacts (loss of jobs), and sustainable mechanisms for doing so probably do not exist. This would also cause social unrest, destroy opportunities for working collaboratively with miners to improve conditions and could simply move illegal mining to other locations.

A third option might be to propose closing ASGM operations that resist becoming legalized Assuming laws are fairly implemented, this could ensure accountability (if the infrastructure existed), but might be directed at particular groups and could further entrench power in the few who are able to obtain licenses, thus making this process unfair. Much would depend on public participation, combined with assistance to support the livelihoods of displaced miners and their families.

#### 2.3.4. Certainty Analysis

Most analysts now believe that developing a fair and open ASGM permit process would have the least economic disruption, could be used to train miners, would produce some tax revenue and no job loss, and could demonstrate cooperation and interest by the government in miners’ future investments and livelihood planning. There is high certainty that such changes would address many of the impediments to regularization, and possibly reduce mistrust of the government. However, implementing such a program would be complicated and costly, and workers will need to be assisted in understanding and complying with the legalization policies [51], while the government ensures improvement in infrastructure to augment ASGM, such as an enhanced road network, reliable electrical power, and safe potable water.

## 3. Conclusions and Implications

Until recently, ASGM has not been recognized as a core component of rural development in most parts of the world [60], implying that policies were often piecemeal, uncoordinated and concerned with short-term solutions to immediate problems. Interventions to help achieve sustainable economic development of rural ASGM communities must first have the welfare of human beings as the focus. This includes the creation of acceptable regulations, but also enforcement of other international norms that prohibit certain kinds of child labor and developing measures to reduce discrimination against women in the various components of ASGM activities [6]. In addition, indicators to assess sustainable rural ASGM communities have not been developed that combine interactions among economic, technological, institutional, and environmental considerations. While these considerations are fundamentally economic, to be sustainable they must have humans and their communities at the core [60]. In a recent report by the U.N. Economic Commission for Africa [6], the Africa Mining Vision describes the goals of “(h)arnessing the potential of ASM to improve rural livelihoods, to stimulate entrepreneurship in a socially-responsible manner, to promote local and integrated national development as well as regional cooperation”. This clear articulation of need summarizes the importance of a long-term, sustainable program of supporting artisanal and small-scale mining, including ASGM, and recognizes its economic importance to individuals, families and nations. 

Interventions aimed at improving the living conditions of people who are part of ASGM communities can only be considered successful if the benefits last for more than the time of interventions. Policies that are designed to be sustainable are needed (Box 1). Most evidence suggests that sector-specific legislation to legalize ASGM will produce many benefits. If undertaken properly, this should enhance community development and economic support, and could be combined with expanded training and use of appropriate technologies [75] that should improve productivity and overall standard of living of mining communities. Effective policy reform is needed that provides consistent and meaningful incentives to people involved in ASGM before environmental protection and sustainability, as well as people's livelihoods, can be improved [73]. Policies do not always define which people are involved in the ASGM sector (as distinguished from large-scale mining operations) and often do not characterize laws and policies in a simple manner that is easily understood by miners and their families. Economic incentives to regularizing ASGM activities are not always clearly articulated, and often do not include accessibility of mineral tenure officials. Using the concepts of “partnership” and “participation”, Childs suggests that bottom–up approaches that involve ASGM members in the creation and implementation of economic and regulatory policies are more likely to be successful and sustainable [76]. 

Financing to improve and develop ASGM represents a possibility for stabilizing and growing this economic enterprise, however credit financing through regular banking systems has been difficult to obtain by most ASGM workers who lack significant collateral. Alternative microcredit or micro-financing programs [74,77] historically have not been very successful either. Recent efforts in the Talensi-Nabdam District of Ghana suggest that more carefully designed programs that meet the needs of ASGM communities might be better received [77].

Box 1 **Summary of policy suggestions that were derived from our Integrated Assessment of evidence concerning social and economic issues of artisanal and small-scale gold mining (ASGM) in Ghana.**

**Summary of Policy Suggestions**Social and economic policies to regularize ASGM activities are likely to be most effective if they are clearly articulated incentives, developing out of "partnerships" with the Minerals Commission, and be easy for miners to understand and follow. Only by assisting miners with means to improve their practices, develop community infrastructure, and employ safer methods will ASGM activities have less environmental impact and greater positive impact on people’s health and well-being. Specific suggestions include:
Interventions to improve the living conditions of people who are part of ASGM communities must be carefully designed to be **sustainable over years and decades**.To improve environmental protection and enhance the health of people involved in ASGM, policies should **provide consistent and meaningful incentives** for miners and their families to follow regulations.If they are to be accepted and effective, policies and laws related to the ASGM sector must be **simple and described in a manner that is easily understood**.To stabilize and grow ASGM as an economic enterprise, **assistance and incentives, including micro-credit and micro-financing,** should be designed and implemented.While protecting the rights of individual miners in the context of social norms, **enforcement of regulations has to be consistent and fair**.To enhance the long-term stability of ASGM economic benefits and human development potential, while minimizing the adverse health and environmental impacts, **constant re-evaluation of policy effectiveness and new needs will be required**.

Sustainable ASGM development will not be easy without support for miners to legalize and formalize their activities. The costs of permit fees, of travel expenses, and of work time lost, contribute to calculations of the perceived value of actually obtaining mineral tenure rights [73]. This makes the transferability of mining rights (mineral tenure) also fundamental to miners’ investing in sustainable and environmentally sound ASGM activities. Mineral tenure can be used, for example, as collateral for financing aimed at developing and sustaining improved mining.

As a complement to this, enforcement of regulations has to be consistent and fair, while protecting the rights of individual miners in the context of social norms. With limited supply enforcement resources, it is important that rules be simple and easily followed if they are to be accepted and respected. Because the necessary governmental infrastructure requires financial support and since gold is constitutionally the property of Ghana, extraction results in a form of taxation. Thus, miners are paid less than the world market price, creating an incentive to evade the government-operated buying system [73]. To improve stability and sustainability of ASGM, the government should be encouraged to enhance capacity to locally certify and track ASGM activities before enforcing sanctions against non-compliant miners [6].

Longer-term economic planning and policy development typically does not consider interactions of ASGM with many other social, environmental and economic considerations. Analytical approaches are needed to help define and implement appropriate policies and actions, at different spatial and temporal scales [78]. Such constant re-evaluation is likely to enhance the long-term stability of ASGM economic benefits and human development potential, while minimizing the adverse health and environmental impacts that need to be addressed.

The effectiveness of these proposed economic and social policies remains speculative because there is relatively little evidence, and most of it comes from programs in countries other than Ghana, e.g., [4,50,74,78,79]. Even less certainty exists regarding the longer-term sustainability of such policy changes, as the ASGM sector is changing rapidly in multiple dimensions. The principles of fairness, openness, and justification of government initiatives have led to longer-term acceptance and compliance in other social domains and economic sectors. This increases the certainty that they can produce stability and sustainability of policy interventions aimed at improving social and economic conditions for ASGM activities. Evidence will be lacking, however, until policy changes such as those introduced through the Minamata Convention [65], are acted upon and outcomes are evaluated over the course of years and decades.
